# Polyaniline Functionalized Peptide Self-Assembled Conductive Hydrogel for 3D Cell Culture

**DOI:** 10.3390/gels8060372

**Published:** 2022-06-13

**Authors:** Jieling Li, Yan Xue, Anhe Wang, Shaonan Tian, Qi Li, Shuo Bai

**Affiliations:** 1 State Key Laboratory of Biochemical Engineering, Institute of Process Engineering, Chinese Academy of Sciences, No. 1 North 2nd Street, Zhongguancun, Beijing 100190, China; jlli@ipe.ac.cn (J.L.); yxue19@ipe.ac.cn (Y.X.); ahwang@ipe.ac.cn (A.W.); 2 University of Chinese Academy of Sciences, Beijing 100049, China; 3 Modular Platform Public Instrument Center, Institute of Process Engineering, Chinese Academy of Sciences, Beijing 100190, China; sntian@ipe.ac.cn

**Keywords:** peptide, self-assembly, polyaniline, conductive hydrogel, cell culture

## Abstract

The functionalization of self-assembled peptide hydrogel is of great importance to broaden its applications in the field of biomedicine. In this work, conductive hydrogel is fabricated by introducing conductive polymer polyaniline into peptide self-assembled hydrogel. Compared with pure peptide formed hydrogel, the conductive hydrogel exhibits enhanced conductivity, mechanical property and stability. In addition, the hydrogel is tested to be of great injectability and 3D bio-printability and could support the viability of encapsulated cells that are sensitive to electrical signals. It should have great application prospects in the preparation of tissue engineering scaffolds.

## 1. Introduction

With the increasing demand for biomaterials in biomedical fields, molecular self-assembly has emerged as a promising strategy for fabricating multifunctional biomaterials [1,2,3]. The molecular self-assembly technique allows the use of relatively simple molecules as building blocks to fabricate nanoscale materials with various morphologies and properties [4,5,6,7,8,9]. Notably, biologically homologous peptides have gradually attracted researchers’ interests as self-assembly building blocks due to their advantages of good biocompatibility, versatility and biological activity [10,11,12]. These peptides have been reported to self-assemble into nanoparticles [13,14], nanotubes [15], nanobelts [16], nanofibers [17] and so on. The inherent sensitivity of peptides to pH, temperature, ions and enzymes provides these self-assembled architectures with good controllability and biological intelligence responsiveness [18,19,20,21]. Furthermore, functional groups could be dopped into peptide assembled architectures to endow them with new functions. In our previous work, we reported the doping of porphyrin into peptide self-assembled nanoparticles to obtain photosensitive nanoparticles. These particles were found to be of great prospect in one and two-photon photodynamic cancer therapy [22,23]. Yan reported the work of photosensitizer modified peptides as building blocks to fabricate nanoparticles with photothermal cancer therapy ability [24]. Among the various architectures, peptide self-assembled fibrous hydrogels are perhaps the most attractive biomaterials for cell culture and tissue engineering due to their tunable mechanical property and fibrous-network structure that can mimic the properties of the natural extracellular matrix. Our group has successfully employed self-assembled peptide hydrogel as a 3D cell culture scaffold for hepatoma culture [25]. Hepatoma could rapidly proliferate within the scaffold into millimeter-sized spheroids, which could serve as a liver cancer model for drug screening and so on. Owing to the tunable mechanical properties and excellent self-healing properties, we have also successfully utilized self-assembled peptide hydrogel as surgical dressings to promote postoperative wound repair [26]. Despite the great progress of peptide self-assembled hydrogel in biomedical applications, it mainly focuses on pure peptide hydrogel, more efforts to fabricate hybrid functionalized peptide hydrogels are urgently required. It is widely known that many biological tissues have their own unique microenvironment to perform their biological functions [27,28,29]. For example, muscle tissue is reported to be in a bioelectric microenvironment, and the function of muscle tissue is controlled by bioelectrical signals [30,31,32]. To mimic the specific microenvironment is of great importance for hydrogels to be used in biomedical fields such as tissue engineering. 

**Scheme 1:**
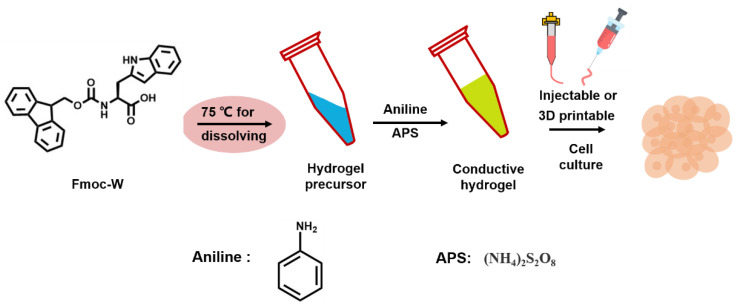
Scheme of the fabrication of conductive hydrogel and its application in 3D cell cutlure.

In this work, As shown in the Scheme 1, conductive polymer polyaniline is introduced into peptide self-assembled hydrogel to fabricate conductive hydrogel. Aromatic peptide Fmoc-W is chosen as the assembly building block. The heated Fmoc-W solution in PBS is found to self-assemble into fibrous hydrogel once cooled to room temperature. For the homogeneous dispersion of polyaniline in the hydrogel, aniline monomers are doped into the hydrogel for in situ polymerization. A certain amount of aniline monomers and APS that served as initiators are added to the heated Fmoc-W solution and mixed thoroughly under shaking before Fmoc-W molecules start gelling. A noticeable color change of the hydrogel from white to brown suggests the successful polymerization of aniline monomers to polyaniline. The π-π stacking between aromatic groups in Fmoc-W and polyaniline molecules could stabilize the hydrophobic polyaniline in the highly hydrated hydrogel, preventing precipitation. The morphology, mechanical property, electrical property, injectability and 3D bio-printability of the polyaniline functionalized peptide hydrogel are characterized. It is suggested that the doping of polyaniline may slightly change the molecular arrangement of peptides, which results in nanofibers of different diameters. The doping of polyaniline not only improves the stability and mechanical strength of the hydrogel but also endows the hydrogel with excellent electrical conductivity, which can be used to conduct electrical signals. Furthermore, the hydrogel also exhibits great injectability and 3D bioprinting performance. The above properties imply that the conductive polymer functionalized hydrogel should be suitable for cell proliferation that is sensitive to the electrical signal. Taking the three-dimensional culture of myoblasts C2C12 as an example, the myoblasts plant in the hydrogel scaffold could quickly proliferate to cell spheroid with a dimension of about 200 μm, and most of the cells in the spheroid survive well, suggesting that the hydrogel could support the three-dimensional cell growth and should have great application prospects in the preparation of tissue engineering scaffolds.

## 2. Results and Discussion

### 2.1. Results

#### 2.1.1. Morphology Characterization of the Hydrogel

**Figure 1:**
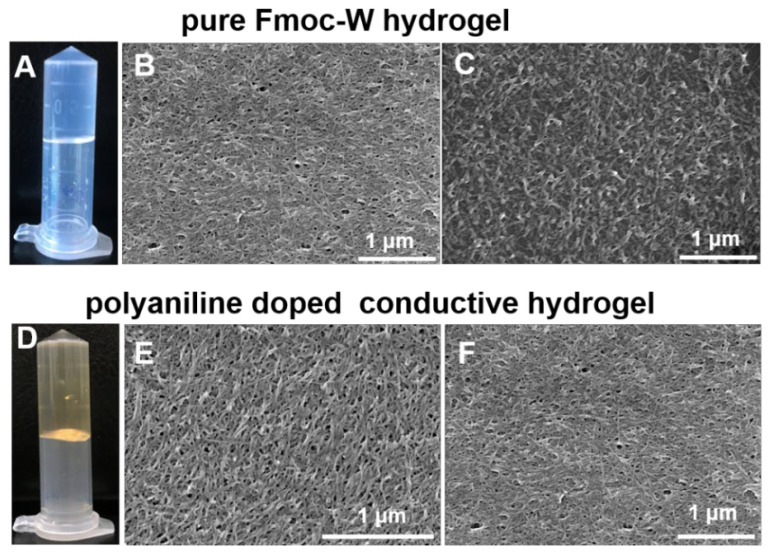
Photo images (**A**) and SEM (**B**,**C**) characterization of pure Fmoc-W hydrogel; Photo images (**D**) and SEM (**E**,**F**) characterization of polyaniline doped conductive hydrogel.

Photo images of obtained hydrogels in Figure 1 showed that pure peptide could assemble into a transparent and colorless hydrogel (Figure 1A), while when doped with polyaniline, yellow–brown transparent hydrogel was obtained (Figure 1D). The morphology of the hydrogels was first characterized by SEM. No apparent morphological difference could be observed where both of the hydrogels were composed of porous structures by interlaced nanofibers (Figure 1B,C,E,F).

**Figure 2:**
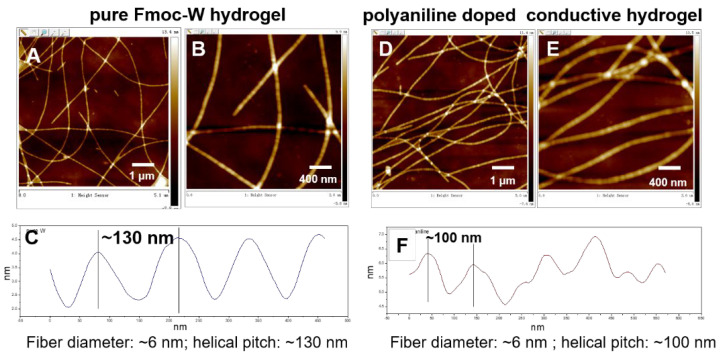
AFM images (**A**,**B**) and helical pitch (**C**) of pure Fmoc-W hydrogel; AFM images (**D**,**E**) and helical pitch (**F**) of polyaniline doped conductive hydrogel.

The detailed morphology of the hydrogels was further observed by AFM. Nanofibrous structures could also be observed for both hydrogels (Figure 2A,D). Magnified images demonstrated helical structures within these nanofibers (Figure 2B,E). The diameter of the nanofibers for pure Fmoc-W hydrogel was measured to be 6 nm and helical pitch to be 130 nm (Figure 2C), and for polyaniline doped hydrogel, the nanofiber diameter was also about 6 nm, while the helical pitch was shortened to be ca. 100 nm (Figure 2F). The results indicated that the addition of polyaniline might slightly change the molecular arrangement during the peptide assembly process.

#### 2.1.2. Mechanical Property and Conductivity Characterization of the Hydrogel

**Figure 3:**
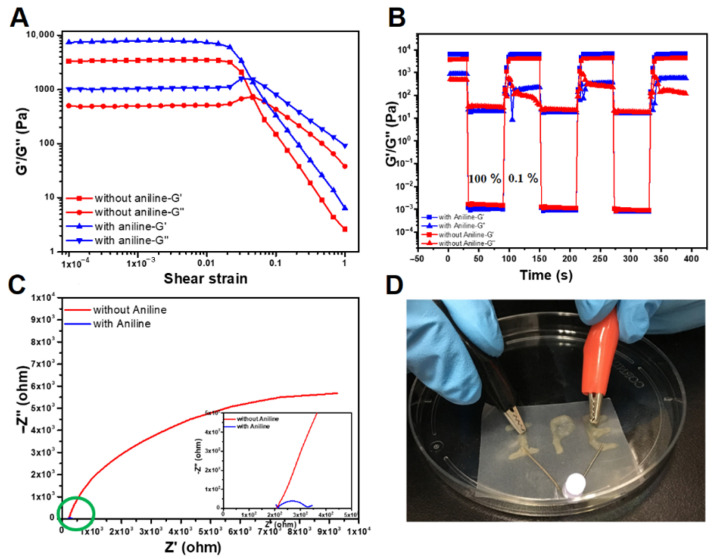
(**A**) Rheological amplitude sweeps of the hydrogels; (**B**) Hydrogels retained the ability to recover their initial viscoelastic properties rapidly (self-heal) following cycles of high strain (high strain: 100%, low strain: 0.1%); (**C**) Nyquist curve of the two kinds of hydrogel, An enlarged view of the green circle is in the lower right corner of (**C**); (**D**) LED illumination test with polyaniline doped hydrogel as conductive wire.

Mechanical property is an important property of hydrogels as it could affect cell behaviors such as adhesion, proliferation, migration and so on. The rheological properties of hydrogels were consequently measured. As shown in Figure 3A and Figure S1, both hydrogels exhibited higher storage modulus (G′) than their loss modulus (G″), demonstrating the typical characterization of elastomer. Notably, the storage modulus (G′) of polyaniline doped hydrogel was much higher than that of pure Fmoc-W formed hydrogel, suggesting that adding polyaniline could significantly improve the mechanical strength of the hydrogel. Furthermore, two hydrogels possessed great shear-thinning (Figure S2) and self-healing properties, as implied in Figure 3B, which means that the storage modulus (G′) of the hydrogels could quickly recover to almost their original G′ after being destroyed. These properties suggested the potential application of the hydrogel as an injectable drug carrier or as 3D bioprinting ink. As polyaniline is a commonly used conductive polymer, we hence proposed that adding polyaniline within the hydrogel could endow the hydrogel with electrical conductivity. We next tested the conductivity of the two hydrogels by electrochemical impedance spectroscopy (EIS). The semicircle diameter in Figure 3C reflected the charge transfer resistance (Rct), where the bigger diameter indicated the greater charge transfer resistance. Obviously, with no polyaniline doping, pure Fmoc-W-formed hydrogel was tested with a quite large semicircle diameter, suggesting the considerable charge transfer resistance of the hydrogel. Comparatively, a hydrogel with polyaniline doping exhibited a relative smaller semicircle diameter, demonstrating a smaller Rct and great electrically conductive ability. To further prove the conductivity of polyaniline doped hydrogel, we next employed the hydrogel as conductive wire for LED illumination. As shown in Figure 3D, when the hydrogel was connected in a closed circuit, the LED could be illuminated, further verifying the outstanding electrical conductivity of polyaniline-doped hydrogel.

#### 2.1.3. Stability and Injectability Characterization of the Hydrogel

**Figure 4:**
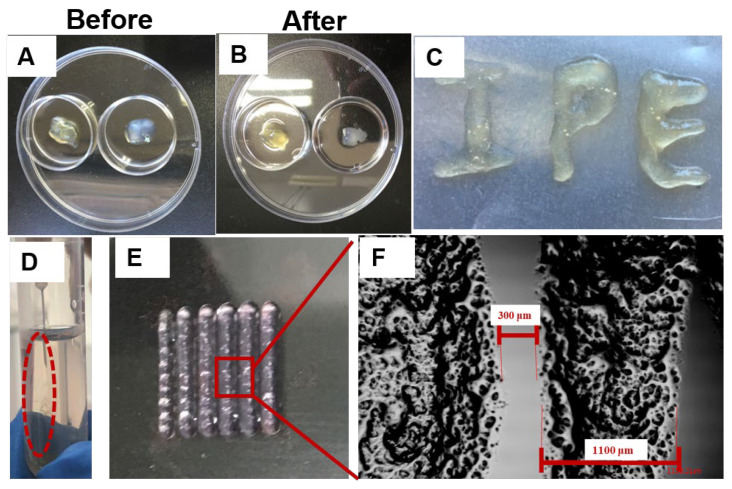
(**A**,**B**) stability characterization of the two kinds of hydrogel; (**C**,**D**) injectability characterization of polyaniline doped conductive hydrogel; (**E**,**F**) printability characterization of polyaniline doped conductive hydrogel.

As for a cell culture scaffold, it was crucial for the hydrogel to be of sufficient stability. Unfortunately, pure peptide-assembled hydrogel often suffered from the disadvantage of instability and was prone to degradation. Here, we wondered whether the addition of polyaniline could improve the stability of the hydrogel. The same amount of two hydrogels was immersed in PBS buffer and incubated at 37 °C for several days. Compared with the original hydrogels in Figure 4A, no apparent hydrogel loss could be observed for polyaniline doped hydrogel (Figure 4B Right), while for pure Fmoc-W assembled hydrogel, significant hydrogel loss was observed, and it showed a clear tendency to degrade (Figure 4B Left). The swelling experiment also showed that the water absorption of polyaniline hydrogel was very low (Figure S3). The different degradation rates suggested that the addition of polyaniline could improve the stability of hydrogels. The mechanical property test proved the great shear-thinning property of the hydrogel, suggesting its injectability and 3D printability. We next verified the injectability by extruding the hydrogel through an injection syringe with a needle of 26-gauge (φ = 260 μm). As shown in Figure 4C, the hydrogel could be continuously extruded from the syringe without any water leaching. Additionally, when injecting PBS buffer with a syringe, the hydrogel could recover immediately to its gel state and be stable enough in buffer solution (Figure 4D), all demonstrating the great injectability of the hydrogel. Furthermore, with the hydrogel as 3D printing ink, we successfully printed parallel hydrogel arrays with a hydrogel wall width of ca 1000 μm and wall space width of 300 μm (Figure 4E,F). The CCK-8 experiment showed that hydrogel had low cytotoxicity (Figure S4). The result suggested the potential application of the hydrogel as a printed scaffold for 3D cell culture.

#### 2.1.4. Three-Dimensional Culture of C2C12 Cells in the Hydrogel

**Figure 5:**
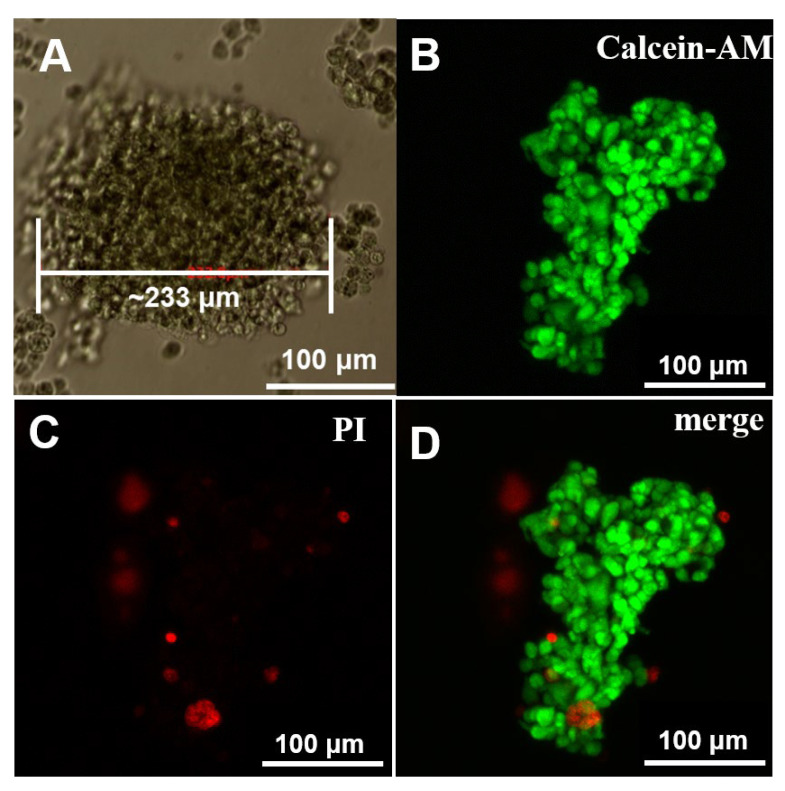
(**A**) photo image and (**B**–**D**) fluorescence images of C2C12 3D culture in polyaniline doped hydrogel scaffold.

Finally, we tested the ability of the hydrogel to support 3D cell culture. As the doping of polyaniline made the hydrogel electrically conductive, we here employed C2C12 as an example cell due to its sensibility to the electrical signal. The conductivity of the hydrogel was supposed to facilitate the signal transition between C2C12 cells and promote cell proliferation. After incubating the hydrogel in DMEM cell culture for three days, C2C12 cells were printed into the hydrogel scaffold by a droplet-based 3D printer and then incubated in DMEM cell culture at 37 °C for three days. Cell proliferation within the hydrogel was firstly observed by an optical microscope (Figure 5A) which showed that C2C12 cells could proliferate into a cell spheroid of about 230 μm in diameter. Next, the obtained cell spheroid was observed by CLSM. Before observation, the cell spheroid was stained with Calcein-AM and PI, which could specifically stain live and dead cells to emit green and red fluorescence, respectively. As shown in Figure 5B–D, cells spheroid with a size of more than 200 μm was observed, and most of the cells in the spheroid were stained green while only a few of the cells were stained red, suggesting that most of the cells were kept alive. The result confirmed the biocompatibility of the hydrogel and implied its potential application in 3D cell culture.

## 3. Conclusions

In this work, conductive polymer polyaniline was introduced into peptide self-assembled hydrogel to prepare conductive hydrogel. The doping of polyaniline not only endowed the hydrogel with excellent electrical conductivity but also improved the stability and mechanical strength without affecting its injectability and 3D bioprinting performance. The hydrogel can act as an active electrical scaffold for the proliferation of cells that are sensitive to the electrical signal. Taking the three-dimensional culture of myoblasts C2C12 as an example cell, the myoblasts planted in the hydrogel scaffold showed a good three-dimensional growth state, indicating that the conductive hydrogel has good biocompatibility and can support three-dimensional cell growth. It may have great application prospects in the preparation of tissue engineering scaffolds.

## 4. Materials and Methods

### 4.1. Materials

Peptide powder, Aniline monomer and ammonium persulfate (APS) were bought from J&K Scientific (San Jose, CA, USA). Dulbecco’s phosphate-buffered saline (PBS) and Dulbecco’s modified eagle’s medium (DMEM) were provided by Gibco (Waltham, MA, USA). Propidium Iodide (PI) and Calcein-AM were purchased from Invitrogen Corp (Waltham, MA, USA).

MCF-7 cell line was obtained from the Cell Culture Center of the Institute of Basic Medical Sciences, Chinese Academy of Medical Sciences (Beijing, China).

The water used in this work was from a Milli-Q Plus water purification system with a resistivity > 18.2 MΩ·cm.

### 4.2. Methods

#### 4.2.1. Hydrogel Preparation

For the preparation of the hydrogel, Fmoc-W powder was first added into PBS buffer (pH 8.0) to form a Fmoc-W suspension. Then, the suspension was heated to 75 °C to promote the dissolution of Fmoc-W powder in PBS buffer. After the powder was completely dissolved in PBS, the obtained clear and transparent solution was cooled to room temperature for the hydrogel formation. To prepare conductive hydrogel, suitable aniline monomer and initiator APS were added to Fmoc-W dissolved PBS buffer before gelling.

#### 4.2.2. Morphology Characterization of the Hydrogel

For scanning electron microscopy (SEM) measurement, an aliquot of the hydrogel was dropped on a silicon wafer and dried in a vacuum at room temperature. Before image acquisition with an S-4800 (HITACHI, Japan, 10 kV voltage) instrument, the sample on a silicon wafer was sputtered with platinum to increase conductivity. 

For AFM characterization, hydrogels were deposited on freshly cleaned mica sheets and dried in a vacuum at room temperature. The images were obtained by scanning the mica surface in air under ambient conditions (FASTSCANBIO, Bruker) and analyzed using the Nano-Scope Analysis software (version 1.5, Bruker).

#### 4.2.3. Mechanical Property

The dynamic rheological properties of the hydrogels were determined by a rheometer (Anton Paar MCR302) with a 12 mm diameter parallel plate (PP12). Strain amplitude sweeps were conducted at a shear-strain (γ) range of 0.01 to 100% and a frequency (f) of 1 Hz. The self-healing properties of the hydrogel were tested by an oscillatory time sweep via alternate strains of 0.1 and 100% (f = 1 Hz). The measurements for each sample were reproduced three times.

#### 4.2.4. Electrical Properties

The electrochemical impedance of the hydrogel was tested with an electrochemical workstation (CHI 660E, Beijing Chinese Science Days Technology Co., Ltd., Beijing, China) using a three-electrode method where the hydrogel, Ag/AgCl electrode and Pt electrode were used as the working electrode, reference electrodes and the counter electrode, respectively. Nyquist curves were measured over the range of 0.01 Hz to 10 kHz at open circuit potential.

#### 4.2.5. Stability of the Hydrogel

The same amount of pure Fmoc-W formed hydrogel and polyaniline doped hydrogel were prepared and incubated in PBS buffer at 37 °C for several days. Then, after removing the PBS buffer, the photo image of the remaining hydrogel was recorded.

#### 4.2.6. Injectability and Printability of the Hydrogel

To test the injectability of polyaniline dopped hydrogel, an extrusion experiment was conducted by extruding the hydrogel through an injection syringe with a needle of 26-gauge (φ = 260 μm).

For 3D printing, the hydrogel was first heated to 50 °C to destroy the hydrogel into liquid. Then, the liquid was printed by a droplet-based 3D printer (CellJet, Thermo Fisher Scientific, Waltham, MA, USA). The printing parameters were optimized as 190 μm for nozzle diameter, 200 nL for droplet volume and 0.9 mm for droplet interval.

#### 4.2.7. Cell Proliferation in the Hydrogel Scaffold

Before cell implanting, the hydrogel was incubated in PBS buffer for three days and then sterilized under UV light for 30 min. After that, 20 μL C2C12 suspension with a density of 10^5^ cell/mL were printed into the hydrogel by a droplet-based 3D printer and incubated at 37 °C for 3 days. To determine the bioactivity of the cells, a Live/Dead staining assay was carried out where Calcein AM (green fluorescence; Invitrogen) and propidium iodide (PI; red fluorescence; Invitrogen) were used for viable and dead cell staining, respectively. The stained samples were observed by a confocal laser scanning microscope (FV1000; Olympus, Tokyo, Japan).

## Data Availability

Not applicable.

## Supplementary Materials

The following supporting information can be downloaded at: https://www.mdpi.com/article/10.3390/gels8060372/s1, Figure S1: Rheological behavior of the hydrogels; Figure S2: Shear thinning test of polyaniline doped conductive hydrogel; Figure S3: Swelling behavior of polyaniline doped conductive hydrogel; Figure S4: CCK-8 experiment with different Fmoc-w concentrations.
