# Supplementary material for: Polyaniline Functionalized Peptide Self-Assembled Conductive Hydrogel for 3D Cell Culture
Source: Gels. 2022 Jun 13;8(6):372. doi: 10.3390/gels8060372 (PMC9222261; doi:10.3390/gels8060372)
Supplement: Supplementary file 1 [file gels-08-00372-s001.zip › gels-1735794-supplementary.pdf]

Supplementary Materials

Polyaniline Functionalized Peptide Self-Assembled Conductive Hydrogel for 3D Cell Culture

Jieling Li [1,†], Yan Xue [1,2,†], Anhe Wang [1], Shaonan Tian [3], Qi Li [1,*] and Shuo Bai [1,*]

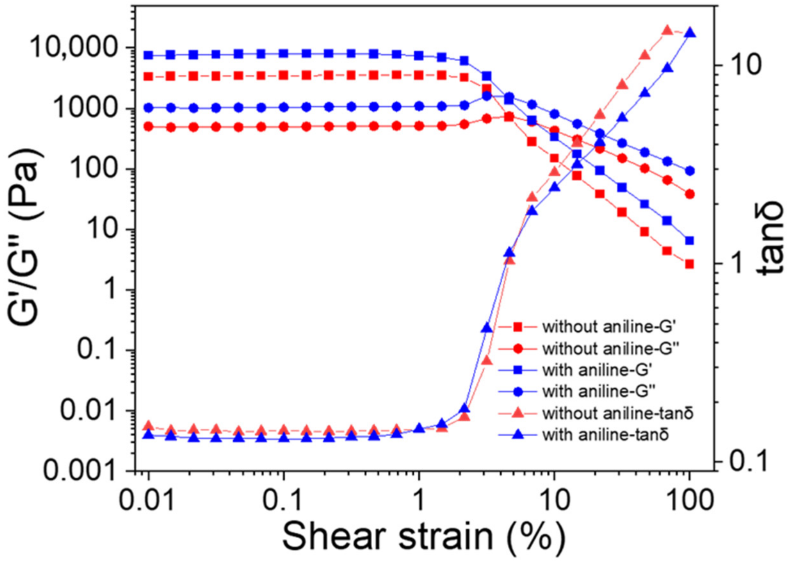

Figure S1. Rheological behavior of the hydrogels.

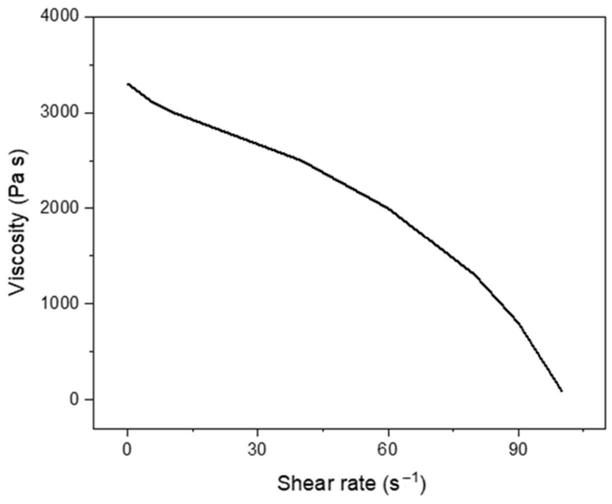

Figure S2. Shear thinning test of polyaniline doped conductive hydrogel.

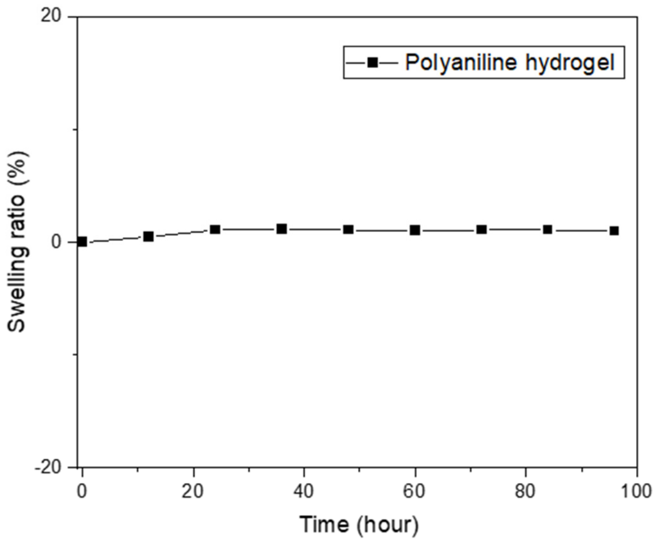

Figure S3. Swelling behavior of polyaniline doped conductive hydrogel.

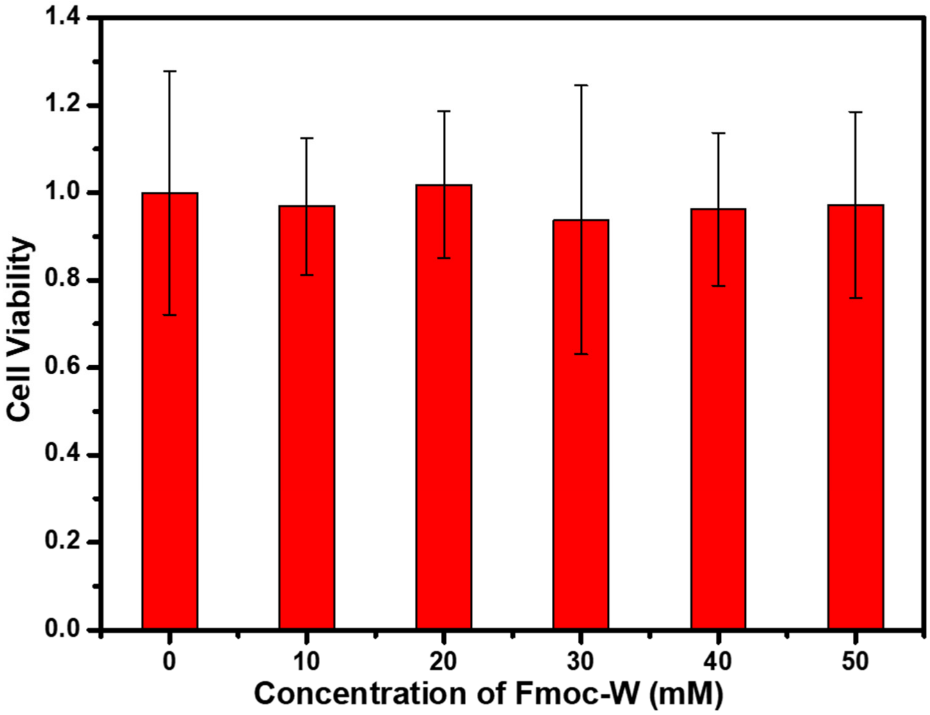

Figure S4. CCK-8 experiment with different Fmoc-w concentrations.
